# Advanced Maternal Age and Adverse Pregnancy Outcome: Evidence from a Large Contemporary Cohort

**DOI:** 10.1371/journal.pone.0056583

**Published:** 2013-02-20

**Authors:** Louise C. Kenny, Tina Lavender, Roseanne McNamee, Sinéad M. O’Neill, Tracey Mills, Ali S. Khashan

**Affiliations:** 1 Department of Obstetrics and Gynaecology, The Anu Research Centre, University College Cork, Cork University Maternity Hospital, Cork, Ireland; 2 School of Nursing, Midwifery and Social Work, University of Manchester, Manchester, United Kingdom; 3 Biostatistics Group of the School of Community-Based Medicine, University of Manchester, Manchester, United Kingdom; 4 NPEC, Department of Obstetrics and Gynaecology, The Anu Research Centre, University College Cork, Cork University Maternity Hospital, Cork, Ireland; 5 The Maternal and Fetal Health Research Centre, University of Manchester, Manchester, United Kingdom; University of Science and Technology of China, China

## Abstract

**Background:**

Recent decades have witnessed an increase in mean maternal age at childbirth in most high-resourced countries. Advanced maternal age has been associated with several adverse maternal and perinatal outcomes. Although there are many studies on this topic, data from large contemporary population-based cohorts that controls for demographic variables known to influence perinatal outcomes is limited.

**Methods:**

We performed a population-based cohort study using data on all singleton births in 2004–2008 from the North Western Perinatal Survey based at The University of Manchester, UK. We compared pregnancy outcomes in women aged 30–34, 35–39 and ≥40 years with women aged 20–29 years using log-linear binomial regression. Models were adjusted for parity, ethnicity, social deprivation score and body mass index.

**Results:**

The final study cohort consisted of 215,344 births; 122,307 mothers (54.19%) were aged 20–29 years, 62,371(27.63%) were aged 30–34 years, 33,966(15.05%) were aged 35–39 years and 7,066(3.13%) were aged ≥40 years. Women aged 40+ at delivery were at increased risk of stillbirth (RR = 1.83, [95% CI 1.37–2.43]), pre-term (RR = 1.25, [95% CI: 1.14–1.36]) and very pre-term birth (RR = 1.29, [95% CI:1.08–1.55]), Macrosomia (RR = 1.31, [95% CI: 1.12–1.54]), extremely large for gestational age (RR = 1.40, [95% CI: 1.25–1.58]) and Caesarean delivery (RR = 1.83, [95% CI: 1.77–1.90]).

**Conclusions:**

Advanced maternal age is associated with a range of adverse pregnancy outcomes. These risks are independent of parity and remain after adjusting for the ameliorating effects of higher socioeconomic status. The data from this large contemporary cohort will be of interest to healthcare providers and women and will facilitate evidence based counselling of older expectant mothers.

## Introduction

The past three decades have seen significant increases in maternal age at childbirth in many high-income countries. [1] The proportion of first births to women aged 35 years and over in the United States increased nearly eight times from 1970 to 2006. In 2006, about 1 out of 12 first births were to women aged 35 years and over compared with 1 out of 100 in 1970. [2], [3] In 2009, the birth rate in the United States declined in all age groups below 40 years but continued to rise in women aged 40–44 and remained unchanged in women aged 45 and over. [4] Similar trends have been observed in Europe. The percentage of live births to mothers aged 35 and over in UK rose from 8.7 in 1990 to 19.3 in 2004 and in the EU-27 the mean age of women at childbirth increased from 29.3 in 2003 to 29.8 in 2009. [5] However, in 2008–2010 it appears that the percentage of live births to mothers aged 35 years and over in England and Wales was stable at about 20% [6].

Advanced maternal age continues to be associated with a range of adverse pregnancy outcomes including low birth weight [7] [8], [9] pre-term birth [7], [8] [10], stillbirth and unexplained fetal death [11]–[13] and increased rates of Caesarean section [14]. However, whilst the volume of literature in this area is impressive, with the majority of studies suggesting an increased risk of adverse pregnancy outcome in advanced age women, some studies have yielded inconsistent conclusions about both the specific outcomes adversely affected by maternal age and the strength of the association [15], [16]. In addition, there is limited consensus among studies as to the precise maternal age when the increase in the risk of adverse pregnancy outcome becomes clinically important. Some studies have reported that the association only becomes significant at age greater than 40 years [17] while others suggest that age ≥35 years is the cut-off for increased risk [18] [10]. These conflicting findings may in part reflect the fact that many of the datasets reported in the literature contain data on births from 25–30 years ago (e.g. [7], [9], [11]). Such data will not reflect recent demographic changes in the antenatal population which may also influence outcome. For example, contemporary older mothers tend to be well educated [19], of higher socio-economic status [20] and of lower parity [5] than older mothers from the recent past. In addition, assisted reproductive technology may have also contributed to the rise in the number of pregnancies in women in their forties. It has been suggested that social advantage may ameliorate some of the adverse effect of advanced maternal age on perinatal outcome. [12], [21]. In recent years, older women who become pregnant are more often primiparous and of better socio-economic status while in the past they were more often multiparous and of low socio-economic status. [21], [22] Moreover, few contemporary studies control for socioeconomic status and other variables, such as body mass index (BMI) and parity that may also influence pregnancy outcome.

Using a large contemporary UK population-based cohort, the present study had 3 aims:

1. To investigate the crude and adjusted associations between advanced maternal age (35 years and older) and adverse pregnancy outcome.
2. To investigate whether any observed associations can be attributed to the confounding effect of known modifiable risk factors (body mass index) or un-modifiable risk factors (ethnic origin).
3. To investigate the association between maternal age and pregnancy outcome in the most and least socially deprived women and in primiparous and multiparous women (subgroup analysis) [16], [21], [22].

## Methods

### Data Source and Study Population

The North Western Perinatal Survey (NWPS), based at St. Mary’s hospital in Manchester, UK collects maternal, infant and obstetrical records on all live and stillborn babies, from 21 maternity hospitals in the North Western region of the United Kingdom (UK) [23]. The data collected by the NWPS covers a large geographic region with diverse social deprivation and ethnicity. In the present study, a cohort study design using the NWPS population-based data from January 1^st^ 2004 until December 31^st^ 2008 was undertaken. The study population included mothers of all singleton babies who were live born or stillborn during the five year study period. Women were categorised according to maternal age into four groups; maternal age 20–29 years (reference category), maternal age 30–34 years, maternal age 35–39 years and maternal age 40 years and older. Women aged less than 20 years were excluded from this analysis. A study on the association between teenage pregnancy and adverse pregnancy outcome in this cohort was published in 2010 [24]. Advanced maternal age in this study is defined as women aged 35–39 years and 40 years and above. Women in the 30–34 year age group were included for completeness.

### Outcome Measures

Advanced maternal age and the following binary pregnancy outcomes were studied: small-for-gestational-age (SGA) and large-for-gestational-age (LGA) babies. Small for gestational age, very SGA (VSGA) and extreme SGA (ESGA), were defined as birthweight below the 10^th^, 5^th^ and 3^rd^ percentile of the gestational age and sex-specific distributions respectively. Large for gestational age, very LGA (VLGA) and extreme LGA (ELGA) were defined as birthweight above the 90^th^, 95^th^ and 97^th^ percentile of the gestational age and sex-specific distributions respectively. For the SGA outcomes (SGA, VSGA and ESGA) the reference group was birthweight ≥10^th^ percentile of the gestational age and sex-specific distributions. For the LGA outcomes (LGA, VLGA and ELGA) the reference group was birthweight ≤90^th^ percentile of the gestational age and sex-specific distributions. Pre-term birth, was defined as any birth before 37 weeks’ gestation and very pre-term birth was defined as any birth before 33 weeks’ gestation. For preterm and very preterm birth analyses the reference group was term babies born at ≥37 weeks’ gestation.

We also examined Macrosomia, defined as a birthweight greater than 4.5 kg, and three Caesarean section outcome measures (all Caesarean deliveries, emergency Caesarean deliveries only and elective Caesarean deliveries only); stillbirth, defined as the birth of a baby without any signs of life after 24 weeks’ gestation and neonatal death defined as death before 28 completed days after birth. Gestational age was based on ultrasound measurements for most babies and on last menstrual period when ultrasound data were not available. Stillbirths were only included in the stillbirths analyses.

### Statistical Analysis

For this analysis, mothers aged 20–29 years of age at time of delivery represented the reference group. Descriptive statistics of baseline maternal characteristics in relation to the maternal age groups were generated. Consequently, crude and adjusted log-linear binomial regression analyses were carried out for each outcome measure separately by maternal age group to estimate the risk ratios (RR). The following potential confounders were included in the adjusted models; BMI (classified into underweight (<18.5 kg/m^2^), normal (18.5–24.9 kg/m^2^) , overweight (25–29.9 kg/m^2^), obese (30–34.9 kg/m^2^) and morbidly obese (≥35 kg/m^2^), parity (primiparous, multiparous), ethnic origin (white, Asian [Bangladeshi and Pakistani], Indian, Black, Chinese, other), and social deprivation score. These factors are known risk factors for various adverse pregnancy outcomes and may be linked with maternal age therefore they are potential confounders. Social deprivation score, determined by postcode, is based on seven deprivation domains (income deprivation, employment deprivation, health and disability deprivation, education deprivation, barriers to housing and services, living deprivation and crime) [25]. For social deprivation, mothers were categorised into four groups, each comprising 22.5% of the population: first group (most deprived), second group, third group, fourth group (least deprived). The fifth group (missing postcodes) comprised 9.2% of the population as data regarding these women’s postcodes were missing. Further adjustment for year of delivery had no material effect on the results therefore it was not included in the final adjusted models. In addition, we adjusted for maternal smoking which was available for the final two years of the cohort as explained later.

We performed subgroup analyses, by stratifying on social deprivation and parity, to assess the association between maternal age and pregnancy outcome in: 1) least socially deprived group and most socially deprived and 2) primiparous and multiparous women. This was done by fitting statistical interaction terms between maternal age and social deprivation and maternal age and parity. To perform the subgroup analyses according to social deprivation we created a new binary variable where the cohort was categorised in two groups; most social deprivation (the first and second group in the original variable described above combined) and least social deprivation (third and fourth groups described above). Women with missing data on social deprivation were excluded from this analysis. The present data had no information on pregnancy loss prior to 24 weeks’ gestation in the population of the North Western Region of England. Therefore the results of the study are conditional on pregnancies that continued to at least 24 weeks. Statistical analysis was performed using SAS 9.2 (SAS Institute Inc, Cary, NC).

### Missing Data

**Figure 1:**
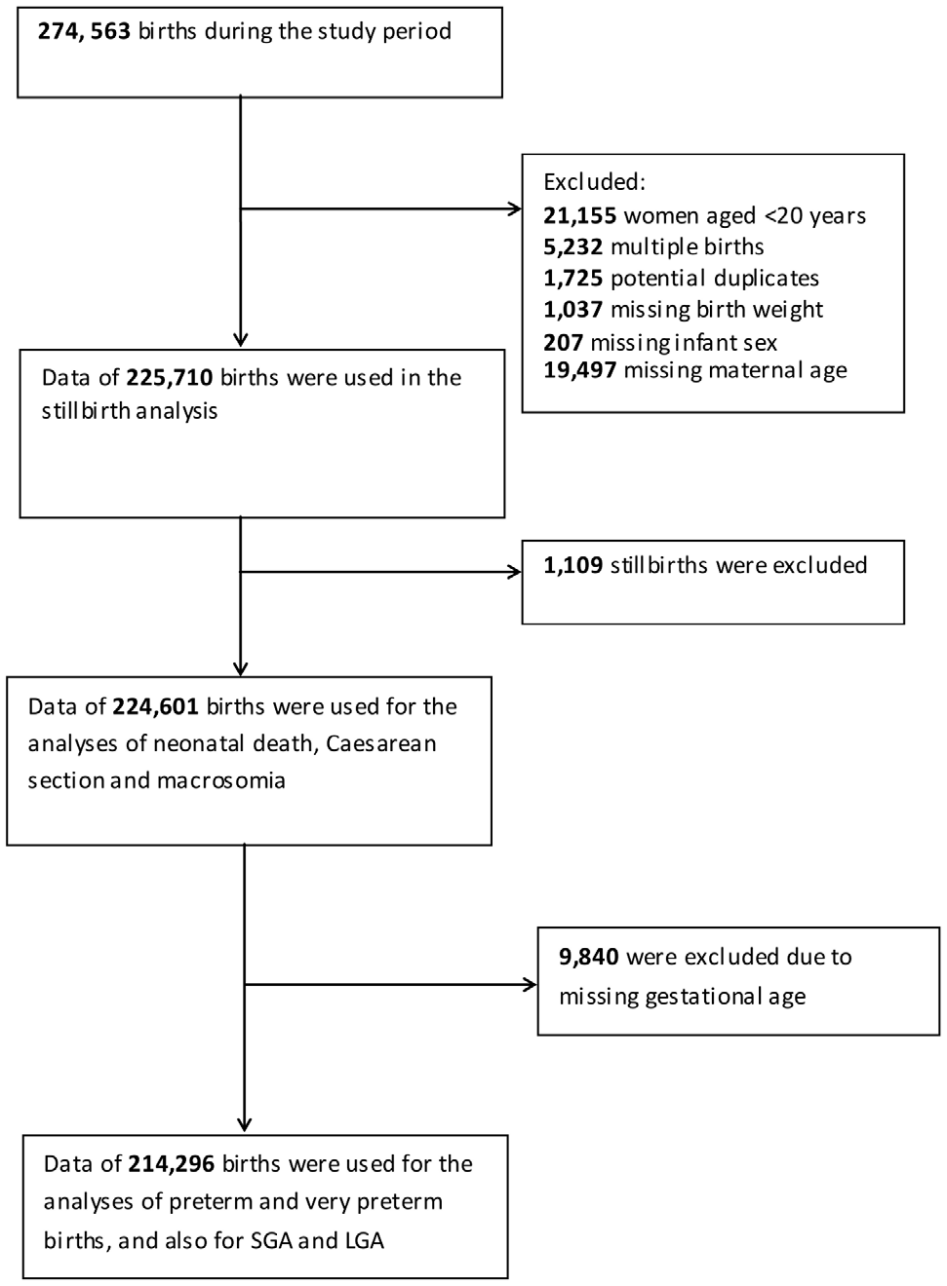
Number of births, inclusions and exclusions during the study period.

Several outcome measures were assessed and so the final cohort for each analysis is dependent on the outcome measure under study. The details of the missing data are presented in Figure 1. The calculation of SGA and LGA requires correct information on birth weight, gestational age and infant sex. Therefore, pregnancies missing data on gestational age at delivery were excluded from the analysis of SGA, LGA as well as preterm delivery outcomes. Maternal smoking data was available for the last two years (2007–08) only. To assess the potential smoking effect on the observed associations, we repeated the analysis by restricting the cohort to offspring of mothers with known smoking status. These models were adjusted for BMI, race, parity, social deprivation score and infant sex. We repeated these models by adding maternal smoking into the model and compared the results to see whether smoking had a confounding effect on any of the associations.

The present study used anonymised data and it was not possible to identify participants. The director of the NWPS gave permission to conduct this study. Thus ethical approval was neither required nor requested.

## Results

During the study period, 274,563 babies were born in the north western region of England. 122,307 women (54.19%) were aged 20–29 years, 62,371 women (27.63%) were aged 30–34 years, 33,966 women (15.05%) were 35–39 years and 7,066 women (3.13%) aged 40 years and older. Data on maternal age was missing for 19,497 (7%) women. On average older women had a better social deprivation score. Sixty eight percent of the population were white, with Asian and Indian women younger, on average, at time of delivery. Multiparous women appeared to be older. Additional descriptive statistics on maternal characteristics and the risk of adverse pregnancy outcome in each age group are summarised in Table 1.

**Table 1:** Maternal characteristics and pregnancy outcome in relation to maternal age.

	Maternal age 20–29 years	Maternal age 30–34 years	Maternal age 35–39 years	Maternal age 40+ years
Maternal Characteristic	122,307 (54.2)	62,371 (27.6)	33,966 (15.1)	7,066 (3.1)
Deprivation score*				
First group: most deprived	34,352 (28.1)	10,786 (17.3)	5,218 (15.4)	1,113 (15.7)
Second group	32,115 (26.3)	11,513 (18.5)	5,737 (16.9)	1,205 (17.1)
Third group	26,910 (22.0)	15,286 (24.5)	8,223 (24.2)	1,738 (24.6)
Fourth group: least deprived	18,354 (15.0)	19,323 (31.0)	11,721 (34.5)	2,350 (33.3)
Fifth group: missing postcodes	10,576 (8.6)	5,463 (8.8)	3,067 (9.0)	660 (9.3)
**Ethnic origin**				
White	80,419 (65.8)	43,137 (69.2)	24,473 (72.1)	4,959 (70.2)
Black	3,647 (3.0)	1,844 (3.0)	975 (2.9)	267 (3.8)
Indian	3,947 (3.2)	1,865 (3.0)	652 (1.9)	86 (1.2)
Asian [ Bangladeshi and Pakistani]	14,372 (11.7)	4,689 (7.5)	1,825 (5.4)	322 (4.6)
Chinese	582 (0.5)	340 (0.5)	172 (0.5)	51 (0.7)
Other	19,340 (15.8)	10,496 (16.8)	5,869 (17.3)	1,381 (19.5)
**Parity**				
Primiparous	50,534 (41.3)	19,334 (31.0)	8,077 (23.8)	1,558 (22.1)
Multiparous	71,773 (58.7)	43,037 (69.0)	25,889 (76.2)	5,508 (77.9)
**Infant sex**				
Male	62,823 (51.4)	32,137 (51.5)	17,356 (51.1)	3,563 (50.4)
Female	59,484 (48.6)	30,234 (48.5)	16,610 (48.9)	3,503 (49.6)
**BMI**				
Underweight	3,262 (2.7)	717 (1.1)	278 (0.8)	63 (0.9)
Normal	44,879 (36.7)	21,043 (33.7)	10,577 (31.1)	1,998 (28.3)
Overweight	23,908 (19.5)	13,857 (22.2)	7,884 (23.2)	1,643 (23.2)
Obese	14,036 (1.48)	7,720 (12.4)	4,525 (13.3)	952 (13.5)
Morbidly obese	1,787 (1.5)	1,001 (1.6)	566 (1.7)	130 (1.8)
Missing BMI	34,435 (28.1)	18,033 (28.9)	10,136 (29.8)	2,280 (32.3)
**Smoking (2007–08)**				
No	38, 515 (72.9)	21,265 (82.4)	12,708 (82.7)	2,849 (80.8)
Yes	11,334 (21.5)	2,985 (11.6)	1,732 (11.3)	444 (12.6)
Unknown (in 2007–08)	2,949(5.6)	1,548(6.0)	931(6.1)	231(6.6)
**Stillbirth**	554 (0.4)	313 (0.5)	190 (0.6)	52 (0.7)
**Neonatal death**	264 (0.22)	144 (0.23)	77 (0.23)	16 (0.23)
**Preterm delivery**	7,041 (6.1)	3,582 (6.1)	2,223(6.9)	464 (6.9)
**Very preterm delivery**	1,528 (1.4)	751 (1.3)	475 (1.6)	100 (1.6)
**All Caesarean sections**	22,424 (18.4)	15,573 (25.1)	9,983 (29.6)	2,397 (34.2)
**Emergency only**	11,839 (9.7)	7,317 (11.8)	4,228 (12.5)	1,016 (14.5)
**Elective only**	10,585 (8.7)	8,256 (13.3)	5,755 (17.0)	1,381 (19.7)
**ESGA**	3,470 (3.2)	1,348 (2.4)	744 (2.4)	169 (2.7)
**VSGA**	5,846 (5.3)	2,286 (4.0)	1,235 (3.9)	281 (4.4)
**SGA**	12,012 (10.3)	4,741 (8.0)	2,444 (7.6)	564(8.4)
**LGA**	10,186 (8.8)	6,995 (11.8)	4,273 (13.3)	906 (13.5)
**VLGA**	4,999 (4.5)	3,567 (6.4)	2,209 (7.3)	496 (7.9)
**ELGA**	2,952 (2.7)	2,162 (4.0)	1,359 (4.6)	298 (4.9)
**Macrosomia**	1,752 (1.4)	1,227 (2.0)	738 (2.2)	162 (2.3)

Data refers to **N (%).** *Birth weight **ESGA** (Extremely small-for-gestational age, <3^rd^ percentile); **VSGA** (Very small-for-gestational age, <5^th^ percentile); **SGA** (Small-for-gestational age, <10^th^ percentile); **LGA** (Large-for-gestational age, >90^th^ percentile); **VLGA** (Very-large-for-gestational age, >95^th^ percentile); **ELGA** (Extremely-large-for-gestational-age, >97^th^ percentile).

The crude estimates of the association between maternal age and adverse pregnancy outcomes are presented in Table 2. The unadjusted logistic models showed that women aged 30 years or more had significantly reduced RRs of ESGA, VSGA and SGA. However, women aged 40 years or older were less protected against SGA than women aged 20–29 years. Increased maternal age was associated with increased RRs of ELGA, VLGA, LGA, Macrosomia, Caesarean section, pre-term birth and stillbirth.

**Table 2:** Crude and adjusted relative risks of the association between maternal age and adverse pregnancy outcome.

	Maternal age, 30–34 years		Maternal age, 35–39 years		Maternal age, 40+ years	
Outcomes	Crude RR (95% CI)	Adjusted ^a^RR (95% CI)	Crude RR (95% CI)	Adjusted ^a^RR (95% CI)	Crude RR (95% CI)	Adjusted ^a^RR (95% CI)
***ESGA (<3^rd^ percentile)***	0.74(0.70, 0.79)	0.91 (0.85–0.97)	0.75(0.69, 0.81)	1.00 (0.92–1.09)	0.83(0.71, 0.97)	1.13 (0.97–1.33)
***VSGA (<5^th^ percentile)***	0.75(0.71, 0.79)	0.90 (0.86–0.95)	0.74(0.69, 0.79)	0.98 (0.92–1.04)	0.82(0.72, 0.92)	1.11 (0.98–1.25)
***SGA (<10^th^ percentile)***	0.75(0.73, 0.78)	0.90 (0.89–0.93)	0.71(0.68, 0.74)	0.92 (0.88–0.97)	0.80(0.73, 0.87)	1.06 (0.97–1.16)
***LGA (>90^th^ percentile)***	1.39(1.35, 1.44)	1.23 (1.19–1.27)	1.59(1.53, 1.65)	1.31 (1.26–1.36)	1.63(1.51, 1.75)	1.32 (1.22–1.42)
***VLGA (>95^th^ percentile)***	1.45(1.39, 1.51)	1.26 (1.21–1.32)	1.68(1.59, 1.87)	1.36 (1.29–1.43)	1.85(1.63, 2.09)	1.44 (1.30–1.58)
***ELGA (>97^th^ percentile)***	1.49(1.41 1.57)	1.30 (1.22–1.38)	1.75(1.64, 1.76)	1.41 (1.32–1.51)	1.75(1.56, 1.96)	1.46 (1.29–1.65)
***Macrosomia (>4.5kg)***	1.38(1.28, 1.49)	1.22 (1.13–1.31)	1.53(1.40, 1.67)	1.26 (1.15–1.38)	1.62(1.38, 1.90)	1.31(1.11–1.54)
***All Caesarean deliveries***	1.36(1.34, 1.39)	1.35 (1.32–1.37)	1.60(1.57, 1.64)	1.59 (1.56–1.62)	1.86(1.79, 1.92)	1.83 (1.77–1.90)
***Emergency Caesarean deliveries***	1.21(1.18, 1.24)	1.28(1.24–1.31)	1.28(1.24, 1.33)	1.41 (1.36–1.45)	1.49(1.40, 1.58)	1.63 (1.54–1.73)
***Elective Caesarean deliveries***	1.53(1.49, 1.57)	1.43 (1.39–1.47)	1.96(1.90, 2.02)	1.77 (1.72–1.83)	2.27(2.15, 2.38)	2.03 (1.93–2.13)
***Preterm delivery (<37 weeks)***	1.00(0.96, 1.04)	1.07(1.03–1.12)	1.15(1.09, 1.21)	1.25 (1.19–1.31)	1.15(1.05, 1.27)	1.24 (1.13–1.37)
***Very preterm delivery (<33 weeks)***	0.96(0.88, 1.05)	1.05 (0.96–1.15)	1.13(1.02, 1.25)	1.25 (1.13–1.40)	1.14(0.93, 1.40)	1.24 (1.01–1.53)
***Stillbirth***	1.11(0.96, 1.27)	1.23 (1.06–1.41)	1.23(1.05, 1.45)	1.41 (1.19–1.67)	1.62(1.22, 2.16)	1.83 (1.37–2.43)
***Neonatal death***	1.07(0.87, 1.31)	1.18 (0.95–1.45)	1.05(0.81, 1.36)	1.18 (0.91–1.54)	1.05(0.63, 1.74)	1.18 (0.71–1.96)

^a^Adjusted for , parity, maternal BMI, social deprivation score and ethnic origin;

b model based on 2007–2008 data only. **ESGA** (Extremely small-for-gestational age, <3^rd^ percentile); **VSGA** (Very small-for-gestational age, <5^th^ percentile); **SGA** (Small-for-gestational age, <10^th^ percentile); **LGA** (Large-for-gestational age, >90^th^ percentile); **VLGA** (Very-large-for-gestational age, >95^th^ percentile); **ELGA** (Extremely-large-for-gestational-age, >97^th^ percentile).

### SGA, LGA and Macrosomia

The adjusted RRs of the association between maternal age and SGA, LGA and Macrosomia are presented in Table 2. There was no evidence to support an association between advanced maternal age and SGA, VSGA or ESGA, with all adjusted RRs close to one and not statistically significant. However, women aged 30–34 appeared to have a significantly reduced RR of SGA (for example ESGA, RR = 0.91, [95% CI: 0.85, 0.97]). Moreover, the RRs of LGA, VLGA and ELGA significantly increased with increasing maternal age compared with the reference group of women aged 20–29 years. For example, the RR of VLGA was increased by 26% (RR = 1.23; [95% CI: 1.21–1.32]) in women aged 30–34 years; by 36% (RR = 1.36; [95% CI: 1.29–1.43]) in women aged 35–39 years; and by 44% (RR = 1.44; [95% CI: 1.30–1.58]) in women aged 40 years and more. The RR for Macrosomia increased significantly with advancing maternal age and again was greatest for women aged 40 years and more (RR = 1.31 [95% CI: 1.11–1.54]). Furthermore, when the analysis was restricted to women with normal BMI, the RR for Macrosomia was increased in women aged 40 years and older by 40% (RR = 1.40, [95% CI: 0.97–2.02]) compared to the reference group although there were a small number of cases (n = 32). For VSGA, VLGA and Macrosomia, BMI, parity and ethnic origin did not appear to have a material confounding effect as none of them changed the estimate by more than 10%, however, social deprivation appeared to change the RRs by more than 10%. When BMI and social deprivation were added to these models together they could explain most of the confounding effect observed between the unadjusted and adjusted models.

### Caesarean Delivery

The RRs of delivery by any Caesarean section (emergency and elective), emergency Caesarean section only and elective Caesarean section only in relation to maternal age are presented in Table 2. Advancing maternal age was significantly associated with an increased RR of all Caesarean delivery outcomes in women of all age groups 30 years or over, when compared with women aged 20–29 years. The highest RR was observed for elective Caesarean section in women aged 40 years or more (RR = 2.03; [95% CI: 1.93–2.13]). The crude and adjusted RRs of Caesarean section (elective and emergency) were similar i.e. the observed association cannot be explained by the confounding effect of BMI, parity, ethnic origin or social deprivation score. Parity partly explained the association between maternal age and elective Caesarean section. Conversely, adjustment for parity appeared to increase the RR of emergency Caesarean section slightly.

### Pre-term and Very Pre-term Delivery

The RR of pre-term and very pre-term delivery increased with increasing maternal age. The results (Table 2) indicated that the RRs of having a pre-term or very pre-term delivery were increased by 24% (RR = 1.25, [95% CI: 1.13–1.37]) and (RR = 1.24, [95% CI: 1.01, 1.53]) respectively in women aged 40 years and older. The adjusted RRs of pre-term and very pre-term birth appeared to be slightly greater, in magnitude, than the crude RRs and most of this increase was related to adjustment for social deprivation score.

### Stillbirth and Neonatal Death

The adjusted RR of stillbirth increased with increasing maternal age and was significantly elevated in women aged 30–34 years, (RR 1.23 [95% CI: 1.06–1.41]) and 35–39 years (OR 1.41 [95% CI: 1.19–1.67]) compared with women aged 20–29 years. Once again, women aged 40 years and older had the highest RR and were almost twice as likely to have a stillborn infant (RR 1.83 [95% CI: 1.37–2.43) compared to the younger reference population. Adjustment for BMI, parity, ethnic origin and social deprivation score appeared to increase the RR of stillbirth in the advanced age groups; however, most of the confounding effect was related to adjustment for social deprivation score. We found very little evidence of an association between increasing maternal age and risk of neonatal death (Table 2).

### Interaction between Maternal Age and Social Deprivation Score

The results (Table 3) showed that the effect of maternal age on emergency Caesarean section and on stillbirth was not dependent on social deprivation score (p>0.05 for all the interaction terms in all age groups). However, women aged 35 years and over were more likely to have an elective Caesarean section if they were in the least socially deprived groups (p<0.05 for the interaction terms in the 35–39 and 40+ age groups). On the other hand, older women in the most socially deprived groups were more likely to have VSGA (30–34 and 40+ years), VLGA (30–39 years) and preterm birth (30–39 years).

**Table 3:** Relative risks of pregnancy outcome and maternal age according to social deprivation group.

	Maternal age 30–34 years	Maternal age 35–39 years	Maternal age 40+ years
Outcome	Adjusted ^a^RR (95% CI)	Adjusted ^a^RR (95% CI)	Adjusted ^a^RR (95% CI)
**Emergency Caesarean section**			
*Most deprived*	1.26 (1.20–1.31)	1.39 (1.32–1.47)	1.61 (1.46–1.78)
*Least deprived*	1.28 (1.23–1.33)	1.42 (1.35–1.48)	1.62 (1.50–1.76)
**Elective Caesarean section**			
*Most deprived*	1.44 (1.38–1.50)	**1.69 (1.61–1.78)**	**1.80 (1.64–1.97)**
*Least deprived*	1.43 (1.37–1.48)	**1.82 (1.74–1.89)**	**2.16 (2.02–2.30)**
**Stillbirth**			
*Most deprived*	1.18 (0.97–1.43)	1.45 (1.14–1.83)	1.67 (1.08–2.60)
*Least deprived*	1.33 (1.05–1.69)	1.41 (1.08–1.86)	2.17 (1.43–3.27)
**VSGA**			
*Most deprived*	**0.96 (0.89–1.03)**	1.01 (0.91–1.11)	**1.25 (1.04–1.50)**
*Least deprived*	**0.80 (0.74–0.87)**	0.89 (0.81–0.98)	**0.90 (0.74–1.09)**
**VLGA**			
*Most deprived*	**1.34 (1.24–1.44)**	**1.48 (1.35–1.61)**	1.52 (1.28–1.81)
*Least deprived*	**1.22 (1.14–1.30)**	**1.29 (1.20–1.38)**	1.38 (1.22–1.57)
**Preterm delivery**			
*Most deprived*	**1.11 (1.04–1.18)**	**1.36 (1.26–1.47)**	1.29 (1.10–1.51)
*Least deprived*	**0.99 (0.93–1.06)**	**1.11 (1.03–1.20)**	1.14 (1.00–1.31)

^a^Adjusted for , parity, maternal BMI, parity and ethnicity; Highlighted estimates indicate a significant interaction test with p<0.05. **VSGA** (Very small-for-gestational age, <5^th^ percentile); **VLGA** (Very-large-for-gestational age, >95^th^ percentile).

### Interaction between Maternal Age and Parity

The results of the statistical interaction between maternal age and parity are presented in Table 4. The results showed that first time mothers were more likely to have emergency Caesarean section (30–39 years), elective Caesarean section (30+ years) and pre-term birth (30–39 years). However, first time mothers aged 30–34 or 35–39 years were less likely to deliver VLGA babies compared to multiparous women.

**Table 4:** Relative risks of pregnancy outcome and maternal age according to parity.

	Maternal age 30–34 years	Maternal age 35–39 years	Maternal age 40+ years
Outcome	Adjusted ^a^RR (95% CI)	Adjusted ^a^RR (95% CI)	Adjusted ^a^RR (95% CI)
**Emergency Caesarean section**			
*Primiparous*	**1.34 (1.28–1.39)**	**1.47 (1.39–1.55)**	1.57 (1.41–1.75)
*Multiparous*	**1.20 (1.15–1.25)**	**1.35 (1.29–1.42)**	1.62 (1.50–1.75)
**Elective Caesarean section**			
*Primiparous*	**1.50 (1.42–1.58)**	**1.98 (1.86–2.11)**	**2.58 (2.32–2.87)**
*Multiparous*	**1.38 (1.34–1.43)**	**1.69 (1.63–1.75)**	**1.88(1.77–2.00)**
**Stillbirth**			
*Primiparous*	1.38 (1.09–1.74)	1.48 (1.07–2.03)	1.82 (0.99–3.35)
*Multiparous*	1.15 (0.97–1.38)	1.37 (1.12–1.67)	1.81 (1.31–2.52)
**VSGA**			
*Primiparous*	0.86 (0.79–0.93)	1.05 (0.94–1.18)	1.03 (0.81–1.30)
*Multiparous*	0.93 (0.87–0.99)	0.95 (0.88–1.03)	1.14 (0.99–1.32)
**VLGA**			
*Primiparous*	**1.14 (1.05–1.25)**	**1.17 (1.03–1.31)**	1.41 (1.12–1.77)
*Multiparous*	**1.31 (1.25–1.39)**	**1.42 (1.34–1.51)**	1.46 (1.31–1.63)
**Preterm delivery**			
*Primiparous*	**1.21 (1.12–1.30)**	**1.50 (1.37–1.64)**	1.42 (1.16–1.73)
*Multiparous*	**1.00 (0.95–1.06)**	**1.15 (1.09–1.22)**	1.17 (1.05–1.31)

^a^Adjusted for , parity, maternal BMI, social deprivation score and ethnicity; Highlighted estimates indicate a significant interaction test with p<0.05. **VSGA** (Very small-for-gestational age, <5^th^ percentile); **VLGA** (Very-large-for-gestational age, >95^th^ percentile).

### Adjustment for Smoking

First the models were performed, using 2007–08 data only, with adjustment for BMI, race, social deprivation score, parity and infant sex. The analyses were repeated by adding maternal smoking into the models (Table 5). These models showed that maternal smoking had a confounding effect on some of the observed associations. The most notable smoking confounding effect was on the RRs of Macrosomia and VSGA where adjustment for smoking reduced the RRs of Macrosomia but increased the RRs of VSGA.

**Table 5:** The confounding effect of smoking on the association between maternal age and adverse pregnancy outcome.

	Maternal age 30–34 years		Maternal age 35–39 years		Maternal age 40+ years	
Outcomes	Adjusted^a^RR(95% CI)	Adjusted^a^RR(95% CI) +smoking	Adjusted^a^RR (95% CI)	Adjusted^a^RR (95% CI) +smoking	Adjusted^a^RR(95% CI)	Adjusted^a^RR (95% CI) +smoking
*VSGA (5^th^ percentile)*	0.89(0.82, 0.96)	0.98(0.90, 1.06)	0.99(0.90, 1.10)	1.11(1.00, 1.22)	1.20 (1.01, 1.43)	1.33(1.11, 1.58)
*VLGA (95^th^ percentile)*	1.25(1.16, 1.34)	1.17(1.08, 1.26)	1.35(1.24, 1.47)	1.26(1.16, 1.37)	1.49(1.29, 1.72)	1.39(1.21, 1.61)
*Macrosomia*	1.25(1.11, 1.41)	1.16(1.03, 1.31)	1.20(1.04, 1.37)	1.10(0.96, 1.27)	1.36(1.07, 1.71)	1.26(1.00, 1.59)
*Emergency Caesarean deliveries*	1.27(1.21, 1.33)	1.26(1.20, 1.33)	1.40(1.32, 1.48)	1.39(1.31, 1.48)	1.77(1.61, 1.95)	1.76(1.60, 1.94)
*Elective Caesarean deliveries*	1.42(1.37, 1.48)	1.40(1.35, 1.46)	1.70(1.63, 1.78)	1.68(1.61, 1.75)	1.89(1.77, 2.02)	1.86(1.74, 2.00)
*Preterm delivery (<37 weeks)*	1.03(0.96, 1.10)	1.08(1.01, 1.16)	1.25(1.16, 1.36)	1.32(1.22, 1.44)	1.20(1.03, 1.39)	1.26(1.08, 1.46)
*Stillbirth*	1.32(1.04, 1.69)	1.39(1.09, 1.78)	1.41(1.05, 1.89)	1.49(1.11, 2.00)	2.03(1.28, 3.20)	2.13(1.35, 3.37)

^a^Adjusted for , parity, maternal BMI, social deprivation score and ethnicity; Highlighted estimates indicate a significant interaction test with p<0.05. **VSGA** (Very small-for-gestational age, <5^th^ percentile); **VLGA** (Very-large-for-gestational age, >95^th^ percentile).

## Discussion

This large cohort study confirms that the risk of a wide range of adverse perinatal outcomes, including stillbirth, pre-term delivery, LGA and Macrosomia increases with increasing maternal age. Older women of higher social deprivation appeared to have a higher risk of fetal outcomes but lower risk of elective Caesarean section compared to women of lower social deprivation. Older first time mothers appeared to have a higher risk of Caesarean section and preterm birth, however, first time mothers aged 30–39 had a lower lower risk of VLGA than multiparous women. The exact mechanism underlying the pathogenesis of adverse pregnancy outcome in older mothers is unclear. It has been suggested that pre-pregnancy obesity and lower socio-economic factors contribute to increase rates of adverse outcomes for women over 35 years of age [26], [27]. In the present study, social deprivation appeared to have a confounding effect on the association between maternal age and several pregnancy outcomes but the confounding effect was limited and did not change the conclusions. However, there was no evidence that BMI could partly explain the association between maternal age and adverse pregnancy outcome.

This cohort has several strengths over other cohorts, including the ability to control for socioeconomic status, parity and BMI. In addition, this contemporary cohort reflects the changing demographics of the older parturient. Older mothers today are more likely to be well educated [19] and of higher socio-economic status [20] compared to their earlier peers who were more likely to be of lower socio-economic status and of high parity. [28] This changing trend in demographics is supported by the present study which shows that older mothers in the north west of England are least likely to be socioeconomically deprived. Higher education and higher socio-economic status are associated with better perinatal and neonatal outcomes, such as term birth and normal birth weight. However, the present study showed that the risk of a wide range of perinatal outcomes including pre-term delivery and stillbirth was elevated even after adjusting for socioeconomic status and BMI. These findings are in accord with data from other high-income populations including Canada [8], Australia [12] and the United States [29].

We also found an incremental and highly significant increase in the rate of both elective and emergency Caesarean delivery in women aged 30 years and more when compared to women aged 20–29 years. This is in line with the findings of other researchers such as Bell et al. who reported Caesarean rates in the range of 25–35% for women aged >35 years and approximately 40% for women aged > 40 years compared with estimates of 14–20% for women aged <35 years. [30].

It has been suggested that trends of Caesarean section for older women appear to be related largely to concerns for fetal welfare [18]. Older primiparous women are most likely to give birth by Caesarean section [10] and recent increases in Caesarean section rates are largely driven by pre-labour Caesarean delivery. [14], [30] This is supported by the findings of the present study which found that primiparous patients over 40 years and older had more than a threefold risk of planned Caesarean delivery. This finding has implications for maternity service providers, particularly as trends of advanced maternal age continue.

The exact age at which adverse outcome for older mothers becomes significant is unclear. Some studies have reported that the association only becomes significant at age greater than 40 years [17] while others suggest that age >35 years is the cutoff for increased risk [18] [10]. By comparing outcomes in all women aged 30 years and more with women aged 20–29 years we were able to demonstrate that the association of adverse outcome with increasing age is a continuum rather than a threshold effect although our comparisons were based on relatively wide age categories.

Several important limitations should be considered when interpreting the results of our study. Firstly, we were able to adjust for maternal smoking, which is independently associated with adverse pregnancy outcome, including an increased risk of stillbirth, for part of the cohort only. Maternal smoking appeared to have limited confounding effect on few associations and it did not seem to change the overall conclusions. This is consistent with the findings of Bahtiyar et al. who also found that adjusting for smoking had no effect on the risk of stillbirth which they reported to be increased in women of advanced maternal age. [31] However, the quality of the smoking variable in this data could be questionnable considering that women were classified as smokers or non-smokers with no information on previous smoking history, whether women quit smoking during pregnancy and how many cigarattes they smoked each day or week. In addition, it is possible that smoking was underreported by pregnant women.

Secondly, we were unable to adjust for co-morbidities such as hypertension and diabetes which have an increased prevalence in older mothers and which are independently associated with adverse pregnancy outcome. However, Cleary-Goldman et al. [29] reported the results from a smaller cohort study of 36,056 women in whom medical history was available. They found older mothers were at increased risk for Macrosomia, pre-term delivery and perinatal mortality in line with the present study and that this risk was independent of maternal co-morbidities. In addition, it could be argued that such co-morbidities are likely to be caused by older age therefore they should not be adjusted for as they are intermediate variables.

Thirdly, we were unable to differentiate between spontaneous pre-term births and medically indicated pre-term delivery. Increased maternal and fetal surveillance of older mothers and real or suspected fetal compromise may provoke aeitrogenic prematurity. This is, at least in part, supported by our observation of increased elective Caesarean section rates in older mothers.

Regardless of the underlying mechanism, the results of this study suggest that contemporary older mothers in the UK are at increased risk of a wide range of adverse pregnancy outcomes and these remain significant after adjustment for several potential confounders including BMI, smoking and socioeconomic advantage. Thus, these findings will be of interest to maternity care providers. The data suggests that increased surveillence of older mothers to detect early signs of adverse pregnancy outcome may be helpful.

### Conclusion

Older mothers are at increased risk of adverse pregnancy outcome compared to their younger peers. This risk is evident in women as young as 30–34 years of age and increases with age. The risk remained high despite adjustment for parity, BMI and socio-economic status. These findings have implications for maternity service providers, particularly as trends of advanced maternal age continue.
